# Contraceptive Counseling for the Transgender Patient Assigned Female at Birth

**DOI:** 10.1055/s-0042-1751063

**Published:** 2022-07-06

**Authors:** Sérgio Henrique Pires Okano, Giovanna Giulia Milan Pellicciotta, Giordana Campos Braga

**Affiliations:** 1 Universidade de Ribeirão Preto, Ribeirão Preto, SP, Brazil; 2 Faculdade de Medicina de Ribeirão Preto, Universidade de São Paulo, Ribeirão Preto, SP, Brazil

**Keywords:** transgender, contraception, testosterone, transgênero, contracepção, testosterona

## Abstract

Although almost 0.7% of the Brazilian population identifies as transgender, there is currently no training for healthcare professionals to provide comprehensive care to these patients, including the discussion of reproductive planning. The use of testosterone promotes amenorrhea in the first months of use; however, this effect does not guarantee contraceptive efficacy, and, consequently, increases the risks of unplanned pregnancy. The present article is an integrative review with the objective of evaluating and organizing the approach of contraceptive counseling for the transgender population who were assigned female at birth. We used the PubMed and Embase databases for our search, as well as international guidelines on care for the transgender population. Of 88 articles, 7 were used to develop the contraceptive counseling model. The model follows the following steps: 1. Addressing the information related to the need for contraception; 2. Evaluation of contraindications to the use of contraceptive methods (hormonal and nonhormonal); and 3. Side effects and possible discomfort associated with the use of contraception. The contraceptive counseling model is composed of 18 questions that address the indications and contraindications to the use of these methods, and a flowchart to assist patients in choosing a method that suits their needs.

## Resumo

Apesar de 0,7% da população brasileira se identificar como transgênera, não existe treinamento para que o profissional de saúde realize um acolhimento de maneira integral a estes pacientes, incluindo a discussão do planejamento reprodutivo. O uso de testosterona promove a amenorreia nos primeiros 6 meses de uso; entretanto, este efeito não garante eficácia contraceptiva, e, consequentemente, aumenta os riscos de uma gravidez não planejada. O presente artigo é uma revisão integrativa com o objetivo de avaliar e organizar uma abordagem do aconselhamento contraceptivo na população transgênera que foi designada mulher ao nascimento. Para a estratégia de busca, foram pesquisados os bancos de dados PubMed e Embase, incluindo diretrizes internacionais sobre cuidados à população transgênera. De 88 artigos, 7 foram utilizados para desenvolver o modelo de aconselhamento contraceptivo. O modelo segue as seguintes etapas: 1. Abordagem das informações relacionadas à necessidade de contracepção; 2. Avaliação das contraindicações ao uso dos métodos contraceptivos (hormonais e não hormonais); 3. Efeitos colaterais e possíveis desconfortos associados ao uso do contraceptivo. O modelo de aconselhamento contraceptivo é composto por 18 questões que abordam as indicações e contraindicações ao uso destes métodos e um fluxograma que auxilia na escolha dentre os métodos permitidos ao paciente de acordo com a sua necessidade.

## Introduction

A transgender person is someone whose gender identity differs from their sex assigned at birth, regardless of their undergoing gender affirmation body procedures. 1 In Brazil, the prescription of hormones for the acquisition of sexual characters of the experienced gender (cross-hormonalization) should be performed according to the guidelines of the Transsexualization Process regulated by Ordinance 2803/2013 of the Ministry of Health, and to the Resolution no. 2.265/19 of the Federal Council of Medicine. 2 3

According to Spizzirri et al. (2021), 4 0.69% (95% confidence interval [CI] = 0.48–0.90) of the Brazilian population identifies as transgender, and 1.19% (95%CI = 0.92–1.47) identify as nonbinary and are of reproductive age (32.8 ± 14.2 years old, 95%CI = 28.5–37.1). Despite these numbers, the population faces several barriers to access health networks due to their lack of visibility. Comprehensive care, which should be based on health promotion, disease prevention, the screening of clinical conditions, and possible treatments, is usually denied to this population, along with reproductive healthcare and, consequently, contraception. 5

People who are assigned female at birth may identify as male or as any other nonbinary gender expression other than female. Transgender men (TM) are understood to be those individuals who have a male gender identity but were assigned female at birth. The sexual orientation of transgender people is independent of their gender identity; that is, TM can relate to people of male, female, and/or nonbinary identities, and can thus be subject to pregnancy when they still have a uterus and ovaries and their fertile sexual partner has a penis. 6 A cohort study identified that transgender people are at potential risk of pregnancy –only 20% of TM used contraception, and more than half were not advised to use contraception after starting hormone treatment. 7 Another American survey showed that 24% of pregnancies in TM occur without planning, mainly due to a lack of guidance on contraceptive methods and the false conception that testosterone has a contraceptive effect. 8

### Surgical Procedures and Contraception

Panhysterectomy and oophorectomy are procedures for patients who desire surgical gender affirmation. Since the organs essential to the reproductive process are removed, there is no need to discuss contraception with the TM who undergoes these surgeries. 9 10 However, family planning by freezing gametes or embryos is a possible pretreatment of fertility and should be offered to all TM prior to commencing hormone therapy and surgery. 5

### Contraceptive Methods

Contraception must be discussed with all people who present a risk of unplanned pregnancy. 7 9 10 11 In Brazil, there is a higher prevalence of the use of oral pills (29.7%) by cisgender women, followed by tubal ligation (14%) and external condoms (10%). 12

Hormonal methods are divided into two groups: combined and progestogen-only contraceptives. 13 14 Hormonal methods act by inhibiting the luteinizing hormone (LH) peak responsible for ovulation. They also alter the cervical mucus to prevent the entry of spermatozoa into the uterine cavity, impair the tubal motility of the fallopian tubes, preventing the egg from moving into the uterine cavity, and alter the endometrial characteristics, making the uterus hostile to implantation. 15 16

Combined contraceptives are a combination of estrogens (either ethinyl estradiol or estradiol valerate) and progestogens, which ensure greater predictability of bleeding, acne control, and hirsutism. 16 However, the presence of estrogen, especially ethinyl estradiol, increases the chances of developing thromboembolic diseases, which limits its use to a restricted group of people. 17 People with breast cancer or those who have been treated for it should not use any hormonal contraception because of the increased risk of recurrence. 18 19

Intrauterine devices (IUDs) do not act through mechanisms based on altering the hypothalamic–pituitary–gonadal axis. In users of copper IUDs, the liberation of ions, which is spermicidal and prevents sperm capacitation, prevents sperm from entering the uterus, while levonorgestrel-releasing intrauterine systems (LNG-IUD) follow the same mechanisms of combined methods, except for the inhibition of the hypothalamic-pituitary-ovarian axis in most users. 20 21 22

### Testosterone Use and Contraception

The use of testosterone promotes the acquisition of secondary male sexual characteristics such as gaining lean mass, muscular hypertrophy, the thickening of the timbre of the voice, and the appearance and growth of hair. 23 Although it is not a contraceptive method, its use is associated with amenorrhea in a massive portion of TM. 9 For this reason, erroneously, 16.4 to 31% of TM believe that testosterone is a contraceptive. 7 10 24

The presence of estrogen in contraceptive methods is one of the factors that leads TM undergoing hormonization to the use of nonhormonal methods or of progestogen alone. 9 10 25 26 Currently, there is no evidence of the influence of contraceptive use on the acquisition of secondary male characteristics. 9 There are also no studies that associate thromboembolism with combined hormonal contraception. 26 Therefore, caution is recommended in the prescription of estrogen due to the lack of data regarding its safety and side effects in the transgender population. 27 The prescription of contraceptive methods should, therefore, follow the same guidelines for contraception in cisgender women proposed by the Center for Disease Control (CDC) and the World Health Organization (WHO). 13 14 28

As such, the purpose of the present review is to evaluate and organize an approach to contraceptive counseling in the transgender population who were assigned female at birth.

## Methods

This is an integrative literature review with the purpose of organizing the existing evidence on the care and particularities of contraceptive counseling in TM, and of the development of an approach model based on this discussion. The search was structured in four axes: indications and contraindications to the use of contraceptives, the use of hormone treatment with testosterone and interference with the use of contraception, side effects of the use of contraception in TM with or without the use of cross-hormone treatment, and possible discomfort related to the method and exacerbation of gender dysphoria.

The PubMed and Embase databases were searched along with the international guidelines on care for the transgender population from the University of California, the International Society of Endocrinology, the World Professional Association for Transgender Heath (WPATH), and the Family Planning Society. 23 25 27 29 30 The keywords *transgender* and *contraceptive* were used to search the databases ( Table 1 shows the search strategy). We considered reviews that were published in the last decade, written in both English and Portuguese, which evaluated contraception in TM, and which evaluated at least one of the outcomes related to contraception in this population. Relevant articles from the bibliographic references of the selected articles were also evaluated as needed.

**Table 1:** Articles used for the literature review

Authors	Year of publication	Study Design	Country	Main Results
Krempasky et al. 9	2020	Review	United States	Transgender male patients deserve the same comprehensive access to care as their cisgender female peers. The most appropriate contraceptive is ultimately the one chosen by the patient. After providing introductory information, subsequent detailed contraceptive counseling should be tailored to the options of their interest. Those who desire contraception should be offered a family-planning consultation in which skilled clinicians may provide the service.
Montoya et al. 31	2021	Review	United States	Understanding contraception, family building, and gender-affirming care are important reproductive health concerns for LGBTQI individuals. Appropriate gender-affirming counseling allows providers to engage in supportive, shared decision-making about contraception with their transmasculine patients. Testosterone therapy is not a substitute for contraception, even when amenorrhea is achieved.
Nisly et al. 32	2018	Review	United States	Nonhormonal intrauterine devices are frequently used; therefore one should avoid not only the hormone effect from hormonal birth control forms but also the perception of the hormone effect, which can be frightening to a transgender man who would not like to have estrogen or progestogen effect. TM fear the effects associated with estrogen or progesterone-containing contraception methods, including reduction of clitoral size or breast enlargement or masculinization reversal.
Gorton et al. 33	2017	Review	United States	Hormone therapy provides significant benefits, allowing many patients to live as male in society by 2 years of treatment. No increased risks of malignancy or cardiovascular end points with hormonal therapy are demonstrated in the literature, although minor increases might not be detectable at the current level of evidence.
Obedin-Maliver et al. 34	2017	Review	United States	If there are no contraindications to combined oral contraception, a continuous low-dose regimen can be prescribed. Levonorgestrel-releasing intrauterine systems are also an option, especially for patients who have a contraindication to, or conceptual aversion to, estrogen-containing compounds. Injectable medroxyprogesterone acetate or daily oral progestin can also be considered for persistent menses.
Mehringer et al. 35	2019	Review	United States	Combined oral contraception can be used for both contraception and menstrual suppression. Combined oral contraceptives are less effective than long-acting contraceptive methods. Estrogen may be perceived to be a “feminine” hormone. Ring may be a less desirable option for some transmasculine individuals who may not identify with the anatomical location of insertion. Depot medryprogesterone acetate is often a popular option for menstrual regulation among transmasculine youth because the progestin has a more androgenic effect compared with other progestins. Intrauterine devices contain no hormones, and, as a result do not provide menstrual suppression. Bleeding patterns with the hormonal intrauterine devices are variable. Bleeding patterns of etonogestrel implants are very unpredictable, and may include irregular prolonged bleeding or spotting, amenorrhea, or increased frequency of bleeding.
Wilczynski et al. 36	2014	Review	United States	Oral progestins (medroxyprogesterone) to decrease androgen secretion. Oral contraceptives can be used to stop menses.

## Results

**Fig. 1.**
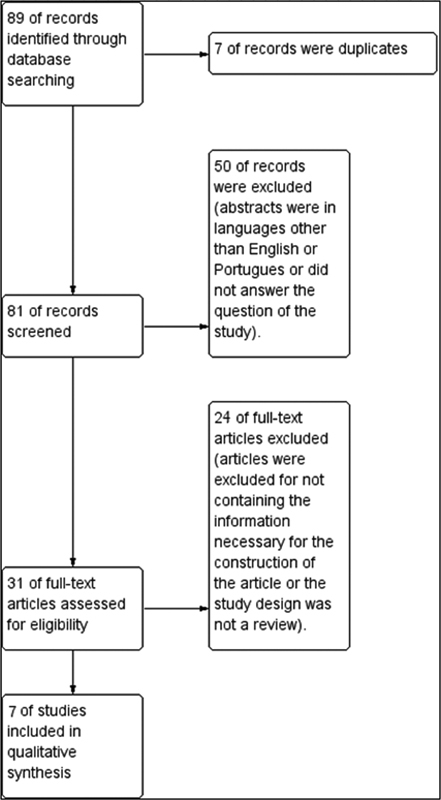
Flowchart.

A total of 88 articles were found, 7 of which were excluded due to duplicity. Of the remaining 81 articles, 51 were excluded for not containing information in the abstract about the study questions, or whose abstract was unavailable or in a language other than Portuguese or English. Twenty-five articles were used for the literature review, and 7 were used to prepare the approach model ( Figure 1 ). The included articles are shown in Table 1 .

## Discussion

The discussion and construction of the approach model was organized in three stages: 1. access to information related to the need for contraception and the noncontraceptive benefits of hormonal methods; 2. evaluation of contraindications to the use of contraceptive methods (hormonal and nonhormonal); and 3. side effects and possible discomfort associated with the use of the method.

### Accessing Information Related to the Need for Contraception and the Noncontraceptive Benefits of Hormonal Methods

Transgender men of fertile age who did not undergo a hysterectomy or oophorectomy and have sexual intercourse with people who have penises are at risk of getting pregnant, even if they are in amenorrhea due to the use of testosterone. 8 Choosing a contraceptive method should be informed by an understanding of the impact of the ovulatory cycle on the quality of life of TM. Complaints such as bleeding, cramps, symptoms of premenstrual tension syndrome (PMS), and pain associated with ovulation can result in physical and psychological discomfort. 5 Symptoms related to the period before vaginal bleeding may exacerbate dysphoric features and should be treated, especially in patients who cannot yet make use of testosterone, such as patients < 16 years old.

### Evaluation of Contraindications to the Use of Contraceptive Methods (Hormonal and Nonhormonal)

All hormonal contraceptives are contraindicated in the presence of a history of breast cancer and hepatocarcinoma. In these conditions, the use of copper IUDs, behavioral methods, spermicides, and barrier methods are acceptable contraceptive options. 18 19 The use of combined methods increases the risk of developing cardiovascular diseases, lithiasis biliary disease, and hypertriglyceridemia. Annex 1 (Supplementary material) shows a model of anamnesis of the contraindications of these contraceptive methods based on the CDC and WHO guidelines for the use of contraceptive methods for cisgender women. 13 14

The research model is composed of 5 core questions, containing a total of 18 “yes/no” subquestions. The first core question seeks to assess the need for contraception of the TM in attendance; any positive response indicates a contraception discussion. The core questions that follow assess the absolute contraindications to contraception, hormonal contraceptive methods, combined hormonal contraceptive methods, and IUD use. A positive response contraindicates their use according to the condition evaluated.

For the preparation of Annex 1 (Supplementary material) , possibilities for the prescription of the clinical conditions presented as categories 1 and 2 of the WHO manuals were considered; the criteria classified as categories 3 and 4 were considered as contraindications to the use of the methods. Conditions or periods such as postpartum were not considered, nor have behavioral and definitive (surgical) methods been discussed in the present article, although they can be oriented to this population. 14

### Side Effects and Possible Discomfort Associated with Contraceptive Use

The choice of contraceptive method should also consider conditions associated with possible discomfort related to the use or insertion/withdrawal of the chosen method. 31 32 33 34 35 36 37 The genital exposure and pelvic manipulation can generate physical, psychological, and emotional discomfort. 6 38

In some protocols, the association of testosterone with an isolated progestogen method at the beginning of therapy is recommended to reduce possible unfavorable bleeding patterns. 8 33 39 Krempasky et al. 9 identified that the reduction or cessation of vaginal bleeding occurs mainly in users of progestogen alone and in users of combined methods on an extended basis (without breaks).

Some medications, such as medroxyprogesterone acetate (DMPA), etonogestrel-releasing subdermal implants, and LNG-IUD may be associated with an increased risk of spotting and bleeding. 40 41 Levonorgestrel-releasing intrauterine systems, despite promoting high contraceptive efficacy and reduced uterine bleeding in the long term, does not block ovulation in most cycles, 22 34 42 thus promoting few benefits related to PMS. Regarding the symptoms related to vaginal bleeding and cramps, except for copper IUDs, all other hormonal methods reduce colic during vaginal bleeding.

Transgender men may present resistance to the use of estrogen in contraception. 26 34 In cisgender women, the use of ethinyl estradiol or estradiol valerate increases the production of sex hormone-binding globulin (SHBG), leading to the reduced bioavailability of testosterone; however, there are no studies that evaluate this effect in transgender people, neither other possible deleterious effects during hormonal transition. 9 31 Some surveys report that the use of exogenous estrogen by this population may occasionally cause the enlargement and development of the mammary glands. 27

Since there are no studies that contraindicate the use of estrogens associated with testosterone in TM, and the risk of developing thrombosis is unknown in this situation, it is prudent to initially offer the prescription of progestogens alone to testosterone users. 10 26 27 The use of combined contraceptives may be offered when there is a desire for or benefit from the association of this hormone for the testosterone-using patient, such as controlling bleeding patterns, acne, or hair loss. 9 35 36 For TM patients who are not on cross-hormonalization, prescribing combined pills with 30 mcg ethinyl estradiol or a vaginal ring in an extended fashion (without breaks) can control unscheduled bleeding by 5 to 25%, while prescribing depot medroxyprogesterone can promote amenorrhea in up to 80% of users. 43 44 45 However, the use of oral pills is associated with femininity, because in cisgender society only women use oral contraceptives; thus, the use of oral contraceptives can be very dysphoric for TM. 27

Depot medroxyprogesterone acetate affects lipid metabolism by reducing all its fractions. 14 46 Two meta-analyses evidenced that the use of testosterone by TM also reduces high density lipoprotein-cholesterol (HDL). 47 48 Despite this side effect, data are still insufficient to assess whether this lipid modification would trigger higher mortality, infarction, strokes, or venous thrombosis in TM. 47 Thus, the association of testosterone with depot medroxyprogesterone acetate should be performed with caution in patients with a significant reduction in HDL.

Annex 2 (Supplementary material) shows a flowchart to assist in the choice of contraceptive methods based on the desire of the patient at three levels – first, addressing the need for contraception, namely, controlling PMS symptoms and vaginal bleeding, or preventing pregnancy; second, evaluating the risks and benefits of this association with testosterone; and third, evaluating the discomfort of the patient with genitopelvic evaluation or manipulation.

This population should not be deprived from the choice of behavioral, barrier, or surgical methods. Transgender men who do not care about bleeding patterns, who have complaints related to their ovulatory cycle, or who simply do not want to use combined contraception may be candidates for other contraceptive methods to prevent unplanned pregnancy.

Although there are other studies that evaluate the need for contraception in TM, 28 the present article suggests an approach model that is yet to be tested. A limitation to the theme is the small number of prospective and randomized studies evaluating the safety of these medications for TM. Studies on contraceptive use and contraindications to methods in the cisgender population may have validity for the transgender population, including in those using cross-hormones with testosterone, although most are observational studies, which limits the degree of evidence of the results.

## Conclusion

Both gynecologists and general practitioners have questions about the particularities of contraceptive prescriptions for the TM population. The use of this objective, semistructured, and easily applied instrument can assist in the discussion and offer of contraceptive methods to transgender patients, and thus improve symptoms associated with the ovulatory cycle and prevent unplanned pregnancies.
