# Supplementary material for: Contraceptive Counseling for the Transgender Patient Assigned Female at Birth
Source: Rev Bras Ginecol Obstet. 2022 Jul 6;44(9):884–90. doi: 10.1055/s-0042-1751063 (PMC9948521; doi:10.1055/s-0042-1751063)
Supplement: Supplementary file 1 — Supplementary Material [file 10-1055-s-0042-1751063-s210468.pdf]

# Annex 1. Addressing contraindications of contraceptive methods for people assigned a female at birth

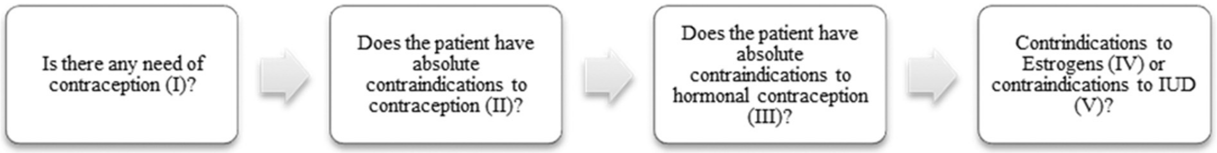


**I. Characterization of the need to use contraception ('yes' indicates the discussion of contraception):**

Do you have vaginal penetrative sexual intercourse with people who have penises? ( ) Yes ( ) No

Do you have PMS symptoms? Are you bothered by the volume or duration of your bleeding, or by the cramping associated with vaginal bleeding?

( ) Yes ( ) No

Do you currently wish to prevent pregnancy?

( ) Yes ( ) No

**II. Assessment of absolute contraindications to contraception:**

Are you pregnant or suspecting pregnancy?

( ) Yes ( ) No

**III. Evaluation of absolute contraindications to the use of hormones:**

Do you have or have you had breast cancer or hepatocarcinoma (liver cancer)?

( ) Yes ( ) No

**IV. Evaluation of relative contraindications: Do you have any of these conditions? ('Yes' contraindicates the use of combined hormonal contraceptives):**

Over 35 years old and smokes?[*]( ) Yes ( ) No

Over 35 years old and diagnosed with migraines?( ) Yes ( ) No

Are you diagnosed with migraines with auras?( ) Yes ( ) No

Multiple risk factors for cardiovascular disease? ( ) Yes ( ) No

*Diabetes Mellitus* with target organ damage?( ) Yes ( ) No

*Diabetes Mellitus* diagnosed more than 20 years ago? ( ) Yes ( ) No

Do you have a diagnosis or suspicion of hypertension? ( ) Yes ( ) No

History of thrombosis and thrombophilia? ( ) Yes ( ) No

Do you use an anticonvulsant other than valproate?[**]( ) Yes ( ) No

Hepatic adenoma?( ) Yes ( ) No

[*]Patients with a smoking load lower than 15 cigarettes/day can use monthly injections.

[**]Patients can use monthly injections, except for lamotrigine users (who cannot use any estrogen).

**V. Evaluation of contraindications to the use of IUD: Do you have any of these conditions? ('Yes' contraindicates IUD use):**

HIV diagnosis with CD4 $<$ 250 cells/$mm^3$? ( ) Yes ( ) No

Disease that distorts the anatomy of the endometrium? ( ) Yes ( ) No

Anatomical alteration or uterine infection?[***]( ) Yes ( ) No

[***]*Information obtained by physical examination in addition to anamnesis.*

 © 2022. Federação Brasileira de Ginecologia e Obstetrícia. All rights reserved.

Annex 2: Decision making regarding the positive and negative aspects of each method

1. What is the purpose of contraception?

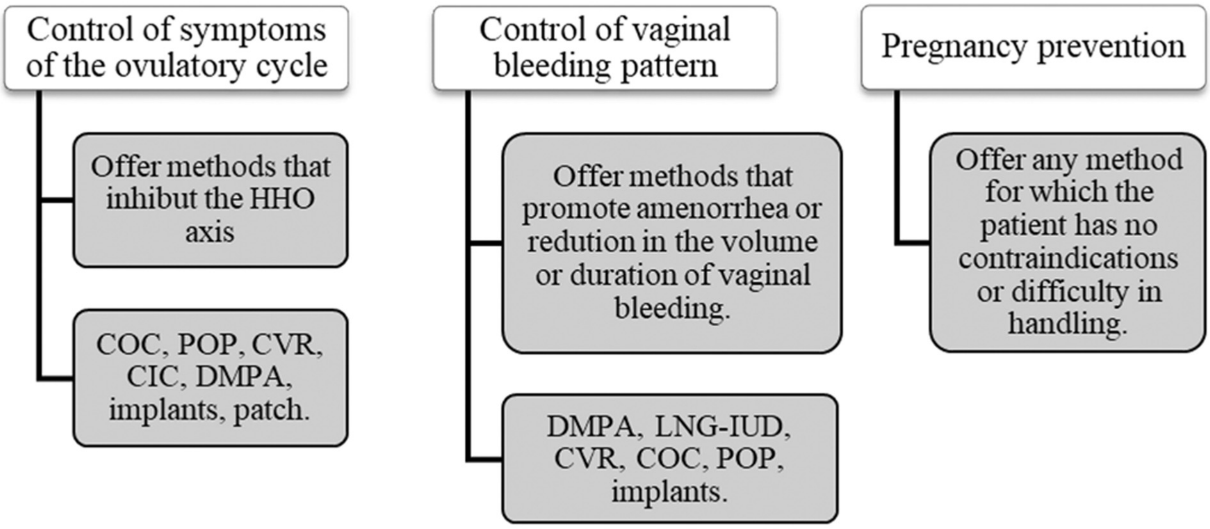


Abbreviations: CIC, combination injectable contraceptives; COC, combined oral contraception; CVR, contraceptive vaginal ring; DMPA, depot medroxyprogesterone acetate; LNG-IUD, levonorgestrel-releasing intrauterine system; POP, progestogens oral pills.

2. Is the patient on testosterone?

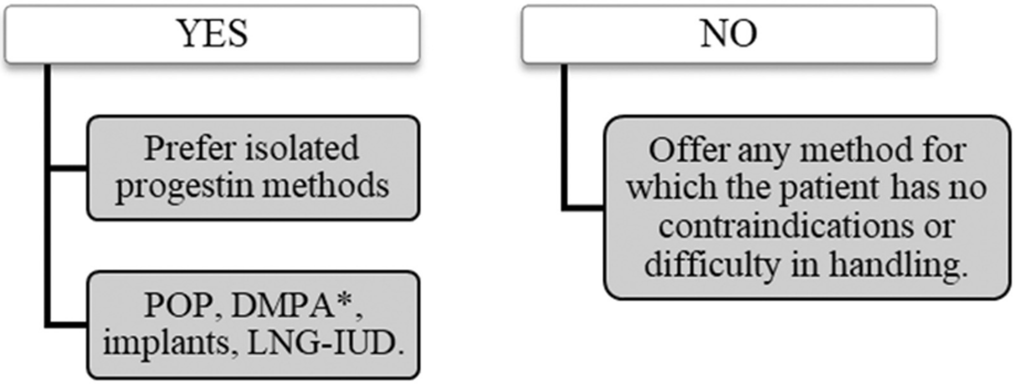


Abbreviations: DMPA, depot medroxyprogesterone acetate; LNG-IUD, levonorgestrel-releasing intrauterine system; POP, progestogens oral pills.

*Pay attention to the association of testosterone with AMP.

 © 2022. Federação Brasileira de Ginecologia e Obstetrícia. All rights reserved.

3. Will the patient be uncomfortable with physical examination or genital contraceptive use?

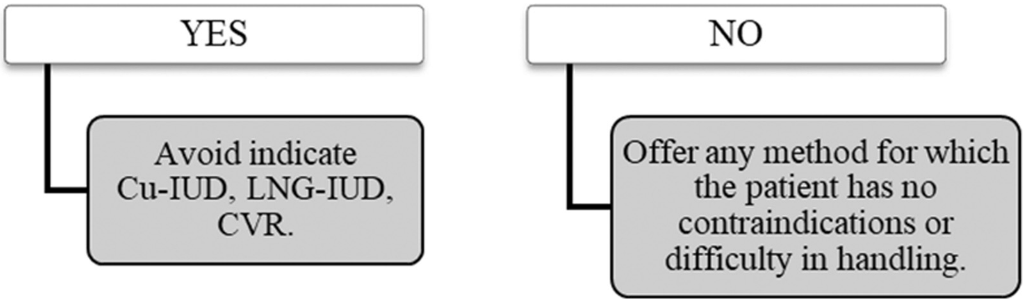


Abbreviations: Cu-IUD, copper intratuterine device; LNG-IUD: levonorgestrel-releasing intrauterine system.

 © 2022. Federação Brasileira de Ginecologia e Obstetrícia. All rights reserved.
